# Acute Stress Induces Different Changes on the Expression of BDNF and trkB in the Mesocorticolimbic System of Two Lines of Rats Differing in Their Response to Stressors

**DOI:** 10.3390/ijms232314995

**Published:** 2022-11-30

**Authors:** Maria Pina Serra, Francesco Sanna, Marianna Boi, Laura Poddighe, Lorenzo Secci, Marcello Trucas, Alberto Fernández-Teruel, Maria Giuseppa Corda, Osvaldo Giorgi, Marina Quartu

**Affiliations:** 1 Department of Biomedical Sciences, Section of Cytomorphology, University of Cagliari, Cittadella Universitaria di Monserrato, 09042 Monserrato, Italy; 2 Department of Life and Environmental Sciences, Section of Pharmaceutical, Pharmacological and Nutraceutical Sciences, University of Cagliari, Cittadella Universitaria di Monserrato, 09042 Monserrato, Italy; 3 Medical Psychology Unit, Department of Psychiatry and Forensic Medicine & Institute of Neurosciences, School of Medicine, Autonomous University of Barcelona, Bellaterra, 08193 Barcelona, Spain

**Keywords:** stress, depression, forced swimming, BDNF, trkB, Roman high- and low-avoidance rats, nucleus accumbens, prefrontal cortex, ventral tegmental area

## Abstract

The present work was undertaken to investigate the effects of acute forced swimming (FS) on the levels of brain-derived neurotrophic factor (BDNF) and tyrosine kinase receptor B (trkB) proteins in: the ventral tegmental area (VTA); the nucleus accumbens (Acb) shell and core compartments; and the anterior cingulate (ACg), prelimbic (PL) and infralimbic (IL) territories of the prefrontal cortex of genetic models of vulnerability (RLA, Roman low-avoidance rats) and resistance (RHA, Roman high-avoidance rats) to stress-induced depression. We report for the first time that FS induced very rapid and distinct changes in the levels of BDNF and trkB proteins in different areas of the mesocorticolimbic system of RHA and RLA rats. Thus, (1) in the VTA and Acb core, FS elicited a significant increase of both BDNF- and trkB-LI in RHA but not RLA rats, whereas in the Acb shell no significant changes in BDNF- and trkB-LI across the line and treatment were observed; (2) in RLA rats, the basal levels of BDNF-LI in the IL/PL cortex and of trkB-LI in the ACg cortex were markedly lower than those of RHA rats; moreover, BDNF- and trkB-LI in the IL/PL and ACg cortex were increased by FS in RLA rats but decreased in their RHA counterparts. These results provide compelling evidence that the genetic background influences the effects of stress on BDNF/trkB signaling and support the view that the same stressor may impact differently on the expression of BDNF in discrete brain areas.

## 1. Introduction

Despite significant advances over the last decades, the etiopathology of depression is still poorly understood. Several hypotheses have been proposed to account for the overall pathophysiological state or core symptoms of depression, based on the dysfunction of monoamine neurotransmission [1], the HPA axis [2] or the neuroimmune system [3]. Another hypothesis posits that the susceptibility to depression results from dysfunction of the mechanisms underlying the plasticity of neuronal networks [4,5] and the abnormal expression of genes that encode neurotrophic factors such as the brain-derived neurotrophic factor (BDNF) and its tyrosine kinase receptor B (trkB) in neural circuits which are modulated by monoaminergic inputs [5,6]. A key tenet of this hypothesis is that the hippocampal expression of BDNF is negatively modulated by stressors and positively modulated by chronic antidepressant treatments [4]; in addition, more recent studies have shown that BDNF/trkB signaling and function in the mesocorticolimbic dopamine (DA) circuits also play a key role in the etiopathology of depression [6].

It is noteworthy, however, that the way by which BDNF is involved in the reactivity to stress in animal models and in the pathogenesis of depression has not yet been precisely established. Thus, the type or severity of stressors may impact differently on the expression of BDNF in discrete limbic brain areas involved in reward- and emotion-related behaviors. For instance, repeated social defeat stress increases both short-term BDNF expression in prefrontal cortical regions and delayed, prolonged BDNF expression in the medial amygdala (AMYG) and ventral tegmental area (VTA) [7]. On the other hand, the local infusion of BDNF in the hippocampus (HC) mimics the behavioral effects of antidepressants [8] but elicits depression-like behaviors if infused in the VTA [9]. This finding suggests that BDNF has an antidepressant effect in the HC but, in contrast, plays a pro-depressant role in the VTA, supporting the view that different molecular mechanisms and neuronal pathways are involved in the effects of BDNF in depression. 

Several animal models have been designed to investigate the impact of the interactions between genetic and aversive environmental factors on the neural substrates of depression. One of these models, the Roman High- (RHA) and Low-Avoidance (RLA) rats, were psychogenetically selected for rapid (RHA) vs. extremely poor (RLA) acquisition of active avoidance in a shuttle-box [10,11]. Subsequent studies have shown that rather than cognitive processes, emotional reactivity is the most prominent behavioral difference between the two lines, with RLA rats being more fearful/anxious and prone to developing stress-induced depression-like behavior than their RHA counterparts, who behave as proactive copers when exposed to stressors [12,13,14]. Interestingly, our recent studies using western blot and immunohistochemistry assays have shown that, under baseline conditions, the expression of BDNF and its receptor trkB is significantly lower in the HC of RLA rats compared with their RHA counterparts [15]. This result is in line with previous studies showing that the availability and/or responsiveness to BDNF are reduced in the HC of depressed patients and of experimental animals exposed to acute or chronic stressful conditions. Of note in this context, we have recently shown that, in RLA rats, an acute stressor such as forced swimming (FS) for 15 min elicits a marked decrease in the level of BDNF protein in the ventral HC (vHC) associated with a significant increase in the dorsal HC (dHC), whereas no significant changes in BDNF levels are observed in either hippocampal compartment of RHA rats [16]. Collectively, these results indicate that multiple factors can determine distinct alterations in the levels of BDNF, including the modality of the stressor, the brain area examined and the genetic background of the experimental subjects. 

Based on the above findings, the present work was undertaken to extend our previous studies in the HC of RLA and RHA rats by investigating the impact of FS on the expression of BDNF and its receptor trkB in the mesocorticolimbic system, using western blot analysis to evaluate their levels and immunohistochemical assays to study their tissue distribution. BDNF-like immunoreactivity (LI) and trkB-LI were measured in limbic areas including the VTA and the shell and core subregions of the nucleus accumbens (Acb), which play a key role in brain reward as well as in the prelimbic/infralimbic (PL/IL) and anterior cingulate (ACg) subregions of the prefrontal cortex (PFC), which is involved in the process of decision-making and shows functional alterations in depressed patients [17,18].

## 2. Results

### 2.1. Behavioral Measures during FS

In keeping with previous studies [16,19,20], the latency to the first immobility episode was significantly shorter in RLA vs. RHA rats. Moreover, the cumulative immobility time was more than two-fold longer in RLA vs. RHA rats. On the other hand, compared with their RLA counterparts, RHA rats spent significantly more time displaying active behaviors like swimming, climbing, and diving. Finally, RHA rats excreted fewer fecal boli than RLA rats (Table 1).

### 2.2. Western Blot

#### 2.2.1. BDNF Protein Levels

**Figure 1:**
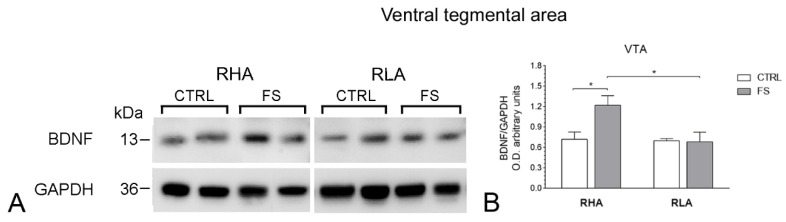
Western blot analysis of brain-derived neurotrophic factor (BDNF) protein levels in the ventral tegmental (VTA) area of RHA and RLA rats under baseline conditions (CTRL) and after a 15 min session of forced swimming (FS): (**A**) shown are representative samples from two rats; (**B**) densitometric analysis of the BDNF/GAPDH band gray optical density (O.D.) ratios. Columns and bars denote the mean ± S.E.M. of seven–eight rats in each experimental group. *: *p* < 0.05 (Tukey’s *post hoc* test or Sidak’s correction for multiple comparisons).

**Figure 2:**
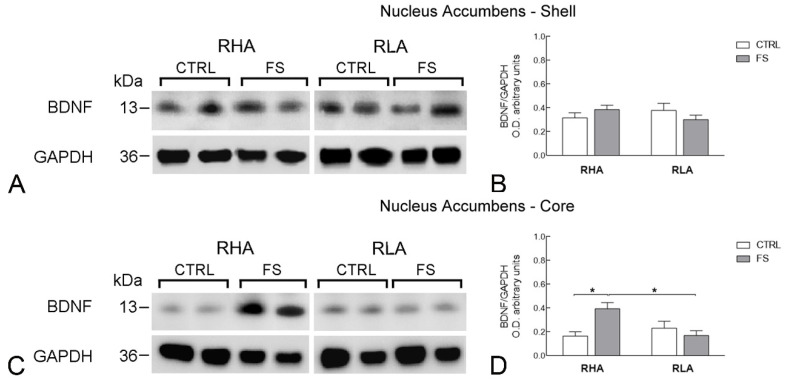
Western blot analysis of brain-derived neurotrophic factor (BDNF) protein levels in the nucleus accumbens shell (**A**,**B**) and core subregions (**C**,**D**) of RHA and RLA rats under baseline conditions (CTRL) and after a 15 min session of forced swimming (FS). (**A**,**C**) shown are representative samples from two rats; and (**B**,**D**) densitometric analysis of the BDNF/GAPDH band gray optical density (O.D.) ratios. Columns and bars denote the mean ± S.E.M. of eight rats in each experimental group. *: *p* < 0.05 (Tukey’s *post hoc* test or Sidak’s correction for multiple comparisons).

**Figure 3:**
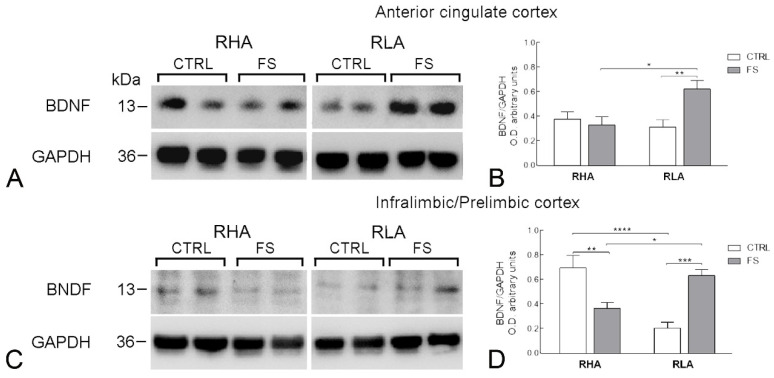
Western blot analysis of brain-derived neurotrophic factor (BDNF) protein levels in the anterior cingulate (**A**,**B**) and the infralimbic/prelimbic (**C**,**D**) regions of the medial prefrontal cortex of RHA and RLA rats under baseline conditions (CTRL) and after a 15 min session of forced swimming (FS); (**A**,**C**) shown are representative samples from two rats; (**B**,**D**) densitometric analysis of the BDNF/GAPDH band gray optical density (O.D.) ratios. Columns and bars denote the mean ± S.E.M. of eight rats in each experimental group. *: *p* < 0.05; **: *p* < 0.01; ***: *p* < 0.001; ****: *p* < 0.0001 (Tukey’s *post hoc* test or Sidak’s correction for multiple comparisons).

The antibody against BDNF recognized one protein band with a relative molecular weight (mw) ≅ 13 kD (Figure 1, Figure 2 and Figure 3), in agreement with the reported mw of the monomeric form of the protein [21].

The statistical evaluation with two-way ANOVA (main factors: rat line and treatment, i.e., FS) of the densitometric values of BDNF-LI in tissue homogenates from the VTA revealed significant effects of line and treatment and also a significant line × treatment interaction (all *p* values < 0.05; Table 2); subsequent pairwise *post-hoc* contrasts showed that, in RHAs but not RLAs, FS increased the BDNF-LI by 70% over the respective control group (Figure 1). 

The statistical analysis of the densitometric values of BDNF-LI in the Acb showed no significant line or FS-related differences in the shell compartment of the nucleus (Figure 2) whereas, in the Acb core, ANOVA revealed a significant line × FS interaction (*p* < 0.01), but no significant effects of line and FS (Table 2; Figure 2). Additional pairwise *post hoc* contrasts aimed at identifying the source of the significant interaction showed that, in RHA rats, FS elicited a large increase of the BDNF-LI value (+137% over the control value, *p* < 0.05) while no significant variation was observed in RLA rats (Figure 2). 

In the medial PFC, the statistical analysis revealed regional differences between the BDNF-LI in the ACg cortex vs. the IL/PL cortex, (Figure 3). Thus, in the ACg cortex, the ANOVA analysis revealed significant effects of line and FS, and a significant line × FS interaction (all *p* values < 0.05; Table 2). Additional pairwise contrasts showed that in RLA rats, but not in their RHA counterparts, FS increased markedly the BDNF-LI value (+98% above the control value; *p* < 0.01; Figure 3). On the other hand, in the IL/PL cortex, FS elicited line-dependent changes in opposite directions on the level of BDNF-LI (Figure 3). Thus, ANOVA showed a highly significant line × FS interaction (*p* < 0.0001; Table 2) and notably, pairwise *post hoc* contrasts revealed that the basal level of BDNF-LI of RLAs was markedly lower (−69%) than that of the RHAs (*p* < 0.0001; Figure 3). Moreover, FS decreased by 47% the BDNF-LI of RHA rats (*p* < 0.01) but increased by 206% the BDNF-LI of RLA rats (*p* < 0.001; Figure 3). 

#### 2.2.2. trkB Protein Levels

**Figure 4:**
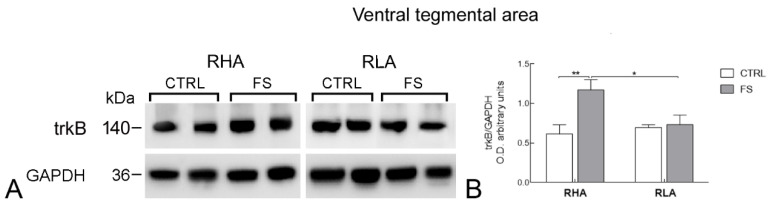
Western blot analysis of the trkB receptor protein levels in the ventral tegmental area of RHA and RLA rats under baseline conditions (CTRL) and after a 15 min session of forced swimming (FS): (**A**) shown are representative samples from two rats; and (**B**) densitometric analysis of the trkB/GAPDH band gray optical density (O.D.) ratios. Columns and bars denote the mean ± S.E.M. of eight rats in each experimental group. *: *p* < 0.05; **: *p* < 0.01 (Tukey’s *post hoc* test or Sidak’s correction for multiple comparisons).

**Figure 5:**
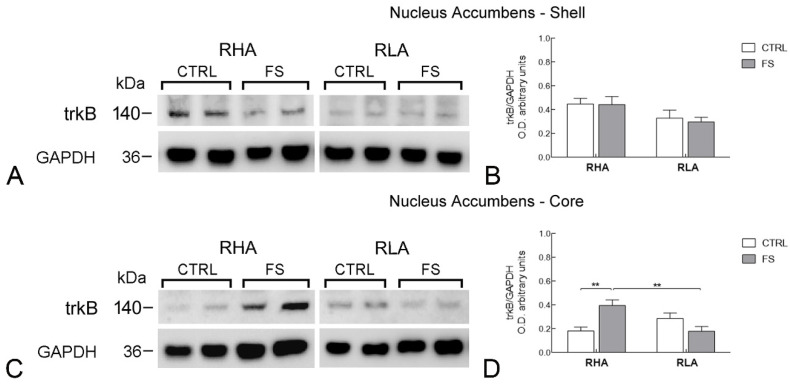
Western blot analysis of the trkB receptor protein levels in the nucleus accumbens shell (**A**,**B**); and core subregions (**C**,**D**) of RHA and RLA rats under baseline conditions (CTRL) and after a 15 min session of forced swimming (FS). (**A**,**C**) shown are representative samples from two rats; and (**B**,**D**) densitometric analysis of the trkB /GAPDH band gray optical density (O.D.) ratios. Columns and bars denote the mean ± S.E.M. of eight rats in each experimental group. **: *p* < 0.01 (Tukey’s *post hoc* test or Sidak’s correction for multiple comparisons).

**Figure 6:**
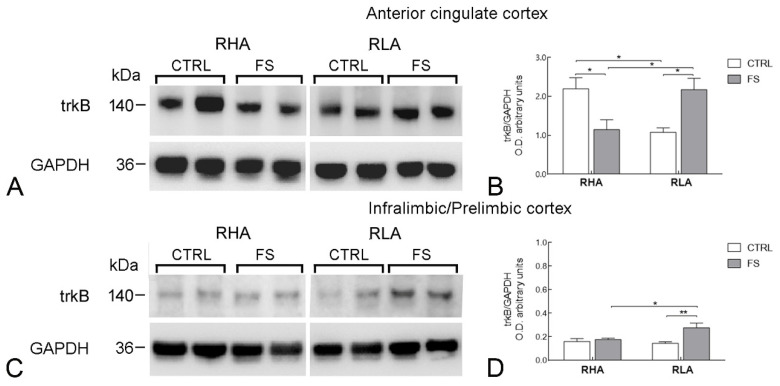
Western blot analysis of the trkB receptor protein levels in the anterior cingulate (**A**,**B**); and the infralimbic/prelimbic (**C**,**D**) regions of the medial prefrontal cortex of RHA and RLA rats under baseline conditions (CTRL) and after a 15 min session of forced swimming (FS). (**A**,**C**) shown are representative samples from two rats; and (**B**,**D**) densitometric analysis of the trkB/GAPDH band gray optical density (O.D.) ratios. Columns and bars denote the mean ± S.E.M. of eight rats in each experimental group. *: *p* < 0.05; **: *p* < 0.01 (Tukey’s *post hoc* test or Sidak’s correction for multiple comparisons).

The antibody against the full-length form of trkB recognized a protein band with a relative mw ≅ 140 kDa (Figure 4, Figure 5 and Figure 6), consistent with the reported mw of the receptor protein [22].

The statistical evaluation of the densitometric values of trkB protein in tissue homogenates from the VTA revealed a significant effect of FS as well as a significant interaction line × FS (both *p* values < 0.05; Table 2). Pairwise *post hoc* comparisons showed that FS increased trkB-LI of RHAs by 90% (*p* < 0.01) but had no significant effect on RLAs (Figure 4). 

The statistical assessment of the trkB-LI in the Acb shell revealed no significant effect of FS on the basal trkB-LI of either line. In the Acb core there was a highly significant interaction line × FS (*p* < 0.001; Table 2) and, in RHA rats, pairwise contrasts showed that FS increased trkB-LI by 117% (*p* < 0.05), whereas no significant change was observed in RLA rats (Figure 5). 

In the medial PFC the statistical analysis of the trkB-LI levels revealed several line-related and cortical subregion-related differences (Figure 6). Thus, in the ACg cortex, there was a significant line × FS interaction (*p* < 0.001; Table 2) and subsequent pairwise contrasts showed that the control trkB-LI value of RLA rats was 51% lower than that of their RHA counterparts (*p* < 0.05); moreover, FS reduced the level of trkB-LI of RHA rats by 48% (*p* < 0.05); in contrast, in RLA rats, FS increased the trkB-LI value by 102% (*p* < 0.05; Figure 6). ANOVA analysis of the trkB-LI values in the IL/PL cortex revealed a significant effect of FS (*p* < 0.05) and a significant interaction line × FS (*p* < 0.01; Table 2). Accordingly, pairwise contrasts indicated that in RLAs, but not RHAs, FS increased trkB-LI by 90% (*p* < 0.01; Figure 6).

### 2.3. Immunohistochemistry

**Figure 7:**
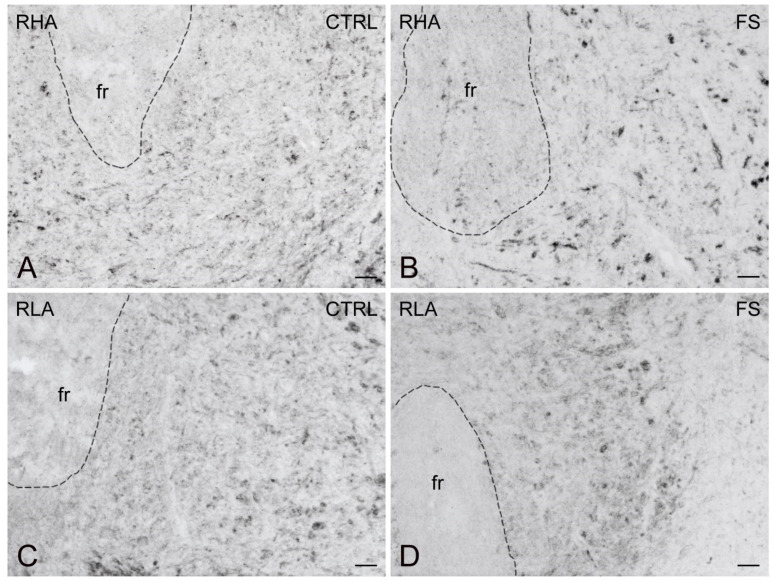
Brain-derived neurotrophic factor-like immunoreactivity (BDNF-LI) in the ventral tegmental area (VTA) of RHA (**A**,**B**); and RLA rats (**C**,**D**) under baseline condition (CTRL) and after a 15 min session of forced swimming (FS). fr, fasciculus retroflexus. Scale bars: 25 µm.

**Figure 8:**
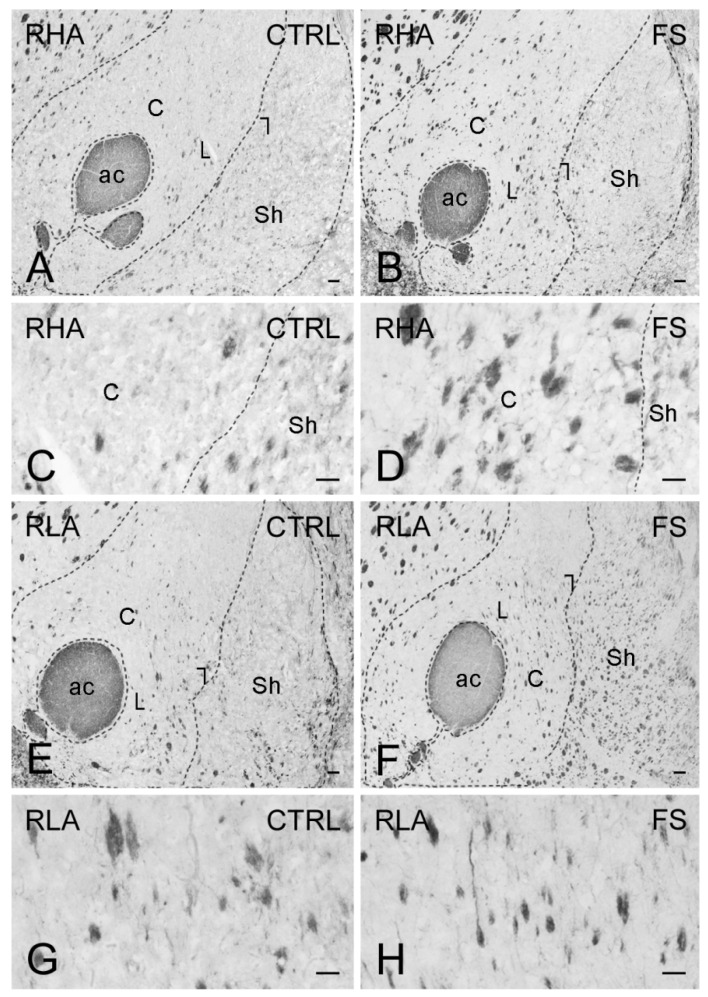
Brain-derived neurotrophic factor-like immunoreactivity (BDNF-LI) in the nucleus accumbens (Acb) of RHA (**A**–**D**); and RLA rats (**E**–**H**) under baseline condition (CTRL) and after a 15 min session of forced swimming (FS). (**A**,**B**,**E**,**F**) low power view of Acb. Dashed lines mark the boundaries of the Acb shell (Sh) and core (C) subregions. Areas squared in (**A**,**B**,**E**,**F**) indicate the microscopic field in (**C**,**D**,**G**,**H**), respectively. ac, anterior commissure. Scale bars: (**A**,**B**,**E**,**F**) = 250 μm; (**C**,**D**,**G**,**H**) =25 µm.

**Figure 9:**
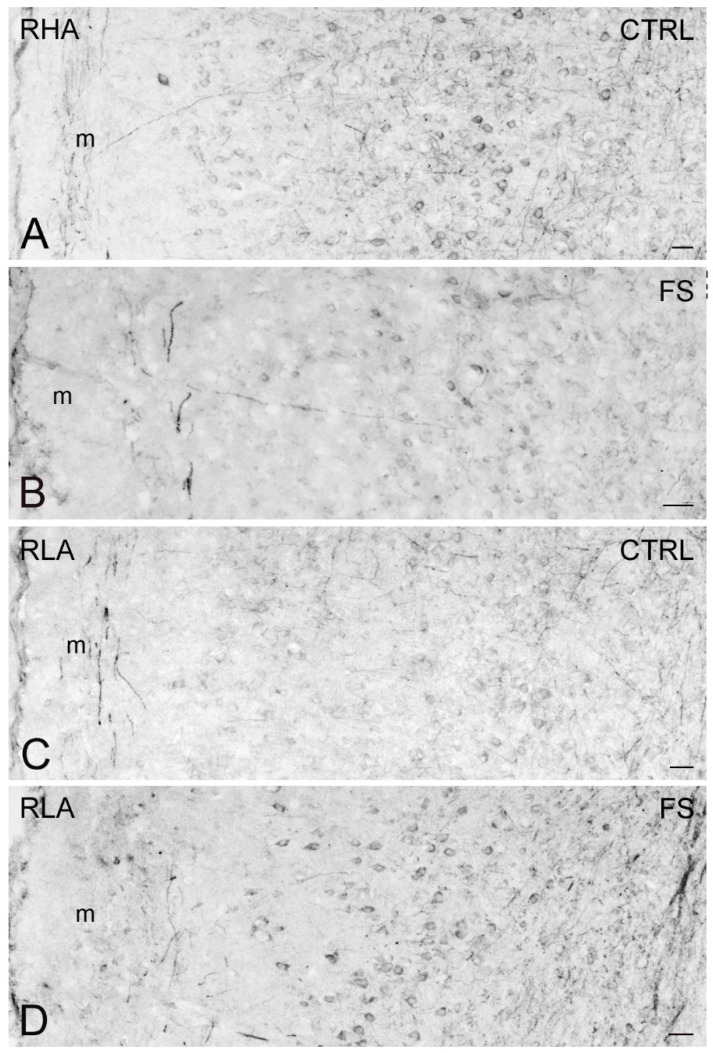
Brain-derived neurotrophic factor-like immunoreactivity (BDNF-LI) in the infralimbic (IL)/prelimbic (PL) region of the medial prefrontal cortex of RHA (**A**,**B**); and RLA rats (**C**,**D**) under baseline conditions (CTRL) and after a 15 min session of forced swimming (FS). m, molecular layer. Scale bars: 25 µm.

**Figure 10:**
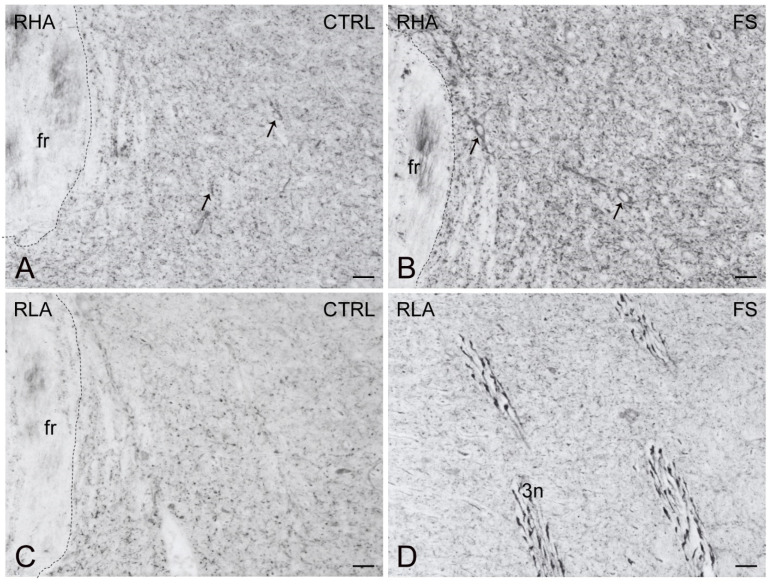
trkB receptor-like immunoreactivity (trkB-LI) in the ventral tegmental area (VTA) of RHA (**A**,**B**); and RLA rats (**C**,**D**) under baseline conditions (CTRL) and after a 15 min session of forced swimming (FS). Arrows in (**A**,**B**) point to labelled perikarya. fr, fasciculus retroflexus; 3n, oculomotor nerve. Scale bars: 25 µm.

**Figure 11:**
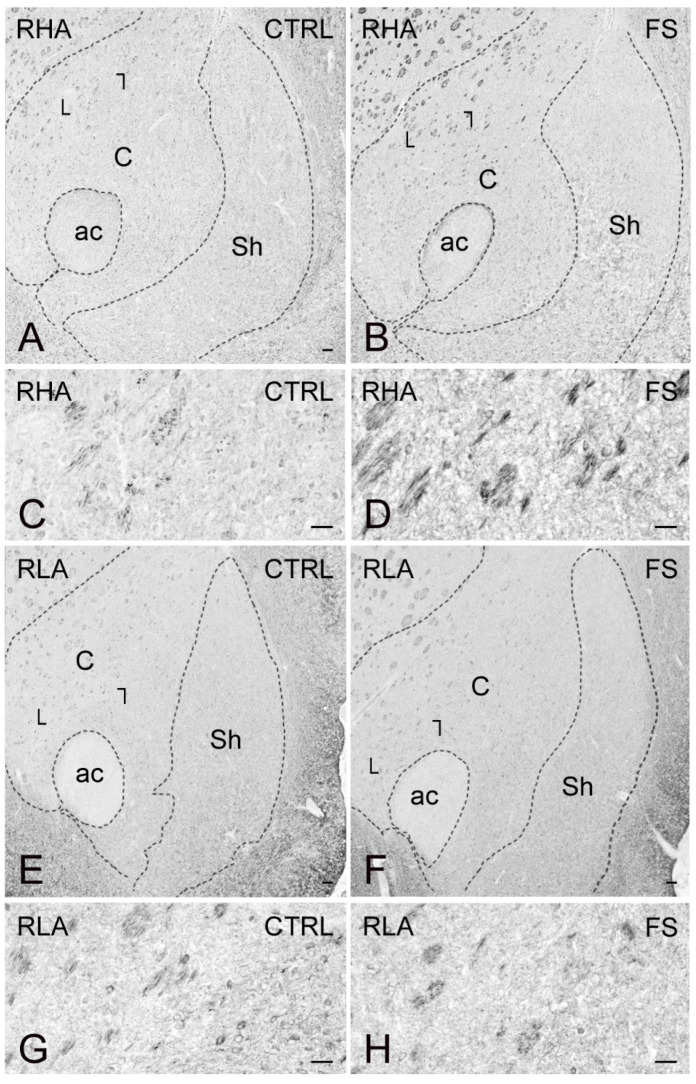
trkB receptor-like immunoreactivity (trkB-LI) in the nucleus accumbens (Acb) of RHA (**A**–**D**); and RLA rats (**E**–**H**) under baseline conditions (CTRL) and after a 15 min session of forced swimming (FS). (**A**,**B**,**E**,**F**) low power view of the Acb. Dashed lines mark the boundaries of the Acb shell (Sh) and core (**C**) subregions. Areas squared in (**A**,**B**,**E**,**F**) indicate the microscopic field in (**C**,**D**,**G**,**H**), respectively. ac, anterior commissure. Scale bars: (**A**,**B**,**E**,**F**) = 250 μm; (**C**,**D**,**G**,**H**) = 25 µm.

**Figure 12:**
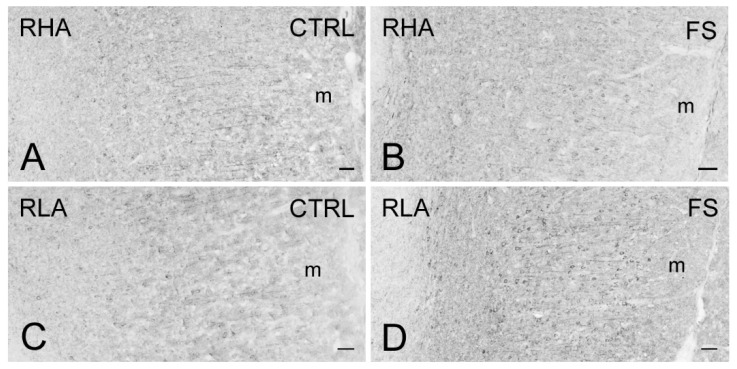
trkB receptor-like immunoreactivity (trkB-LI) in the infralimbic (IL)/prelimbic (PL) region of the medial prefrontal cortex of RHA (**A**,**B**) and RLA rats (**C**,**D**) under baseline conditions (CTRL) and after a 15 min session of forced swimming (FS). m, molecular layer. Scale bars: 25 µm.

Immunohistochemical assays were performed in slices from the VTA, Acb and the ACg and IL/PL subregions of the medial prefrontal cortex to further define the anatomical and cellular distribution of the BDNF- (Figure 7, Figure 8 and Figure 9) and trkB-LI (Figure 10, Figure 11 and Figure 12) at an optical microscopic level. 

Immunostaining for BDNF occurred in neuronal perikarya, proximal neuronal processes, and in thread-like elements, interpreted as nerve fibers and appearing either isolated or as nerve bundles, loose networks, and varicosities in proximity to unstained cell bodies (Figure 7, Figure 8 and Figure 9). In the VTA territory, BDNF-LI was present in cell bodies and in sporadic dot-like structures (Figure 7); several short nerve fiber tracts, often transversely sectioned and likely representing extrinsic nerve fibers crossing the region, were also observed, particularly after FS in RHA rats (Figure 7B). In the Acb, BDNF-like immunoreactive nerve fibers, mainly appearing as darkly stained, transversely sectioned bundles were observed in the core compartment while the Acb shell displayed loose meshes of isolated filaments (Figure 8). Rare BDNF-immunolabeled neuronal cell bodies were also present. In the medial PFC, differences could be appreciated in the aspect of BDNF-LI; thus, except for the molecular layer, positive neuronal perikarya were the prevailing elements throughout the cortical layers of RHA rats in basal conditions and in both rat lines after FS (Figure 9); in contrast, BDNF-like immunoreactive nerve fibers were predominant over positive perikarya in control RLA rats (Figure 9).

As shown in Figure 10, Figure 11 and Figure 12, the trkB-LI was localized to neuronal perikarya, proximal neuronal processes and nerve fibers. In the VTA, trkB-labeled large neuronal perikarya and dot-like elements were observed in RHA rats, particularly after FS (Figure 10). In both the shell and core compartments of the Acb, trkB-positive neurons stood out within a diffuse labeling of the neuropil (Figure 11). Except for the molecular layer, numerous trkB-labeled neuronal cell bodies and long neuronal processes were observed in all the layers of the IL/PL (Figure 12) and ACg (not shown) subregions of the PFC. 

## 3. Discussion

The present results show for the first time that BDNF and trkB are differentially expressed and distinctly regulated by stress in the mesocorticolimbic system of RHA and RLA rats; thus, (1) in the VTA and Acb core, FS elicited a significant increase of both BDNF and trkB-LI in RHA but not RLA rats whereas in the Acb shell no significant changes in BDNF- and trkB-LI across line and treatment were observed; (2) in RLA rats, the basal levels of BDNF in the IL/PL cortex and of trkB in the ACg cortex were markedly lower than those of RHA rats; moreover, BDNF- and trkB-LI in the IL/PL and ACg cortex were increased by FS in RLA rats but decreased in their RHA counterparts.

### 3.1. RLA and RHA Rats Display Different Coping Strategies in Response to FS

RLA and RHA rats represent two divergent phenotypes displaying respectively reactive and proactive coping styles in the face of aversive environmental conditions [12,13,14]. Notably, the more fearful/anxious RLA rats are also more prone than RHA rats to develop a stress-induced depression-like phenotype that is normalized by chronic treatment with antidepressant drugs [19,20], thus adding experimental evidence supporting the key role of both stressful environmental conditions and genetic background in the etiology of depression. The results of the present study confirm and extend previous behavioral data [16,19,20] demonstrating that, during an acute 15 min session of FS, RLA rats exhibit longer lasting immobility and shorter swimming, climbing, and diving times when compared to their RHA counterparts. 

Preclinical and clinical gene expression and imaging studies support a neurotrophic hypothesis of depression and antidepressant response. This hypothesis proposes that depression results from decreased BDNF-mediated support leading to neuronal atrophy, decreased neurogenesis and loss of glia in the HC, and that antidepressant treatment blocks or reverses this BDNF deficit [23,24]. 

Because depression is often precipitated or exacerbated by acute or chronic stressful life events [25,26] and stress can cause damage and atrophy of neurons in certain brain structures, different stressors are frequently used in preclinical models of depression to study alterations of brain structure and function. Notably, the predominant observation across diverse stress paradigms is a decline in the expression of the BDNF gene as well as BDNF protein levels and signaling in the HC and PFC [27,28,29]. Accordingly, we have previously reported that the basal BDNF levels are lower in the HC of RLA vs. RHA rats [15]; in addition, in the present study we show that the basal BDNF- and trkB-LI are lower in RLA vs. RHA rats in the IL/PL and ACg cortex, respectively. Together, these findings suggest that the basal BDNF/trkB signaling is weaker in the HC and PFC of rats that are prone to developing a stress-induced depression-like phenotype as compared to depression-resilient rats.

### 3.2. Role of the BDNF/trkB System and the Dopaminergic Mesocorticolimbic Pathways in Stress-Induced Depression-Like Phenotypes

While it is well established that the HC and frontal cortex are involved in aspects of depression and its treatment [29,30], other neural circuits in the brain, more closely related with emotions, also play a key role in this mental disorder. Thus, a growing body of experimental evidence derived from more recent studies indicates that the mesocorticolimbic dopamine (DA) pathway may contribute markedly to the pathophysiology and symptomatology of depression [31]. Moreover, significant advances have been made in the identification of the molecular, cellular, and neural circuit mechanisms in the mesocorticolimbic DA systems involved in the ability of stressors to induce depression-relevant behavioral and functional phenotypes. These studies focused on the role of BDNF and trkB-mediated signaling in the murine mesolimbic (VTA → Acb) and mesocortical (VTA → PFC) DA pathways, most frequently using as stress models the chronic social defeat stress (CSDS) [6,32] and the chronic unpredictable stress (CUS) [33] paradigms.

Animals subjected to CSDS (one daily defeat over 10 days for mice and one defeat every 3 days for 10 days for rats) display a long-lasting reduction in social interaction, a behavior reminiscent of social withdrawal in depressed patients [7,34,35]. This depression-like phenotype is associated with an increment in BDNF signaling, but not DA transmission, in the VTA and Acb shell of mice [6] and an increase in BDNF protein and mRNA expression in the PFC of rats [7]. This prodepressant role of mesocorticolimbic BDNF signaling is in direct contrast to the antidepressant-like actions of BDNF in the HC, which emphasizes the circuit-specific nature of molecular mechanisms involved in stress-induced depression.

Notably, very different changes in DA and BDNF signaling in the mesocorticolimbic system are seen with the CUS paradigm [36]. Animals exposed to CUS (a series of physical stressors for 1–12 weeks [33]) display abnormal behaviors that are considered as measures of anhedonia, such as a decrease in sucrose preference and social interaction [33]. This depression-like phenotype is associated with decreased activity of VTA → PFC DAergic neurons in vivo as well as reduced levels of BDNF in the PFC tissue and in the supernatant of PFC slices in vitro (the latter being considered as an indicator of the amount of secreted BDNF) [36]. In line with the decreased activity of the mesocortical DA transmission in animals submitted to CUS, we have previously shown that tail pinch stress and anxiogenic drugs are able to elicit a significant increment in the release of DA in the PFC of RHA but not RLA rats suggesting that the functional tone of the mesocortical DA projection is less intense in depression-prone RLA rats vs. their resilient RHA counterparts [14]. Moreover, the above findings support the view that, in the CUS paradigm, the decreased firing rate of VTA → PFC DAergic neurons and the reduction of BDNF content and secretion in the PFC have prodepressant effects while the increase in DAergic neuron firing rate and BDNF signaling in the PFC have antidepressant effects [36]. Of note, neither DA nor BDNF signaling in the VTA → Acb DAergic neurons appear to play a role in these effects, which underscores the stress paradigm-specific nature of the molecular, cellular and circuit mechanisms involved in stress-induced depression-related behaviors [36].

### 3.3. Differential Effects of Stress on BDNF and trkB Expression in the Mesolimbic Pathway of RLA and RHA Rats

The effects on BDNF/trkB signaling in the mesolimbic pathway induced by acute FS in the Roman rats are at variance with those observed with chronic stress paradigms, i.e., CSDS and CUS, in rodents. Thus, because RLA rats, but not their RHA counterparts, are predisposed to displaying depression-like behaviors when exposed to FS [19,20], we predicted that acute stress would elicit more pronounced changes in BDNF and trkB protein levels (either an increase or a decrease) in the mesolimbic DA system of RLA vs. RHA rats. Conversely, acute FS induced a significant increase in the levels of both, BDNF and trkB only in the VTA and Acb core of RHA rats which are resilient to stress-induced depression-like behavior [14,19,20]. Instead, no significant changes were observed upon FS in the Acb shell of either Roman line. 

These results are consistent with the hodological and functional differences between the core and shell subregions of the Acb and with the evidence that selective manipulations of these accumbal subregions produce distinct functional alterations due to the differential balance of cortical and limbic inputs to these two subregions [37,38]. Thus, the Acb core is considered to play a role in guiding goal-directed behavior, whereas the Acb shell seems to be crucial for the control of reward-seeking behaviors [37,38,39,40]. Such functional differences have been reported to be associated with a diverse regulation of DA release in the two subregions [39,41]. Therefore, it may be proposed that the increment of BDNF/trkB signaling in the VTA and Acb core of RHA rats represents an adaptive neuroplastic response to the prodepressant effect of FS while BDNF/trkB signaling in the Acb core of RLA rats has a marginal role in the behavioral response to FS displayed by this line. Whether the selective increase of BDNF signaling in the mesolimbic circuit of RHA rats is a molecular mechanism underlying the characteristic proactive coping behavior displayed by this Roman line when exposed to aversive conditions [42] remains to be established.

### 3.4. Differential Effects of Stress on BDNF and trkB Expression in the Mesocortical Pathway of RLA and RHA Rats

Our results indicate that the basal levels of BDNF/trkB-LI are lower in the PFC of depression-prone RLA rats vs. their RHA counterparts. This finding is in line with the results reported by Liu et al. [36] using the CUS paradigm in mice and with several reports in humans [29,30] and references therein. 

In view of the propensity of RLA rats to display a depressive phenotype when exposed to stressors we expected that acute stress would elicit more pronounced changes in BDNF/trkB signaling in the mesocortical system of RLA vs. RHA rats. Accordingly, FS induced a significant increase in the levels of both, BDNF and trkB, in the ACg and PL/IL cortices of RLA rather than RHA rats. In keeping with our results, in rats subjected to CSDS, BDNF protein and mRNA expressions were increased in the PFC [7]. Importantly, because the levels of BDNF and trkB proteins remained unchanged in the VTA of RLA rats, the source of the increments in the levels of BDNF and trkB was most probably elsewhere. In this context, it is likely that the sources of BDNF and trkB were glutamatergic projection neurons and GABAergic interneurons of the PFC, respectively [31]. By the same token, because FS elicited an increase in the levels of both BDNF and trkB in the VTA of RHA rats, it appears unlikely that the VTA was involved in the decrease in the levels of BDNF in PL/IL cortex and trkB in ACg cortex of this Roman line.

The PFC sends projections to the hypothalamus and the periaqueductal grey, which mediate the visceral motor activity associated with emotion, and the Acb, which signals reward and motivational value [29]. Anatomical and functional studies have shown that the ventral aspect of the IL/PL cortex provides excitatory glutamatergic inputs to the Acb shell while its dorsal aspect together with the ACg cortex send excitatory projections to the Acb core and the caudo-putamen (see for review [14]). In turn, the Acb shell sends inhibitory GABAergic input to the DAergic neurons of the VTA. These neural connections mediate the control of the midbrain DA system by the ventral part of the medial PFC [43,44]. 

Here we have shown that FS is able to elicit in the PFC of RLA but not RHA rats an increment of the expression of both BDNF and trkB in the IL/PL and ACg cortices. Further studies are warranted to establish whether the alterations of BDNF/trkB transmission elicited by FS in the medial PFC of RLA rats are either signaling depression-like phenotypes to subcortical structures or instead represent neuroplastic adaptations of cortical neurons to face the load of the acute stress.

### 3.5. Final Remarks

Collectively, the results reviewed above raise the question of how the same stress paradigm can induce divergent modifications of BDNF/trkB signaling in different circuits of the mesocorticolimbic system. The answer may lie within the anatomical and functional heterogeneity of the VTA: a growing number of studies provide compelling evidence that the VTA is heterogenous in terms of anatomy, neurochemical profile, electrophysiology, and afferent/efferent connectivity [45]. VTA neurons have typically been classified as principal (primarily dopaminergic ~65%), secondary (GABAergic ~30%), or tertiary (other, mostly glutamatergic ~3%) based on immunohistochemistry for tyrosine hydroxylase, as well as electrophysiological and pharmacological properties [46,47,48]. Importantly, one major source of BDNF in the VTA and its projection brain areas is provided by glutamatergic afferents from medial PFC, HC and AMYG, which express high BDNF levels. BDNF released from these afferents acts on trkB receptors expressed on VTA, Acb and PFC neurons. VTA DAergic neurons express moderate levels of BDNF, and this may provide an additional source of BDNF in the VTA and Acb [31]. In contrast, Acb neurons express very low levels of BDNF, and the physiological role of this source remains unclear. 

Electrophysiological studies have shown that a rewarding stimulus activates VTA DAergic neurons projecting to the medial zone of the Acb shell whereas an aversive stimulus activates DAergic cells projecting to the medial PFC. In contrast, VTA DA neurons projecting to the lateral zone of the Acb shell are activated by both rewarding and aversive stimuli, which presumably reflects saliency. These results suggest that the mesocorticolimbic DA system may be comprised of at least three anatomically distinct circuits, each modified by distinct aspects of motivationally relevant stimuli [49]. 

In this context, it is important to mention that RHA and RLA rats, bidirectionally selected for their distinct coping strategies in the two-way active avoidance paradigm (proactive in RHAs vs. reactive in RLAs), display marked differences in the function of mesocorticolimbic DA transmission. Thus, when subjected to tail pinch stress, RHA rats exhibit a proactive coping strategy associated with a marked increment in DA output in microdialysates from the PFC; conversely RLA rats display reactive coping behavior and do not show increased DA output in the PFC, suggesting that the functional tone of the mesocortical pathway is stronger in RHA vs. RLA rats [14]. Furthermore, RHA rats show a more intense locomotor activation in response to addictive substances like psychostimulants and morphine [41], as well as a higher preference for ethanol [50] and palatable food (our unpublished results), and more intense sexual motivation than RLA rats [51]. All these different responses are associated with a larger DA output in the Acb shell of RHA vs. RLA rats [51,52,53]. Finally, only RHA rats display behavioral sensitization following the repeated administration of psychostimulants and morphine, and this effect is associated with a larger DA output in the Acb core of RHAs vs. RLAs, suggesting a more intense functional tone of the mesolimbic pathway of RHA rats [41]. Therefore, it is plausible that the different characteristics of the mesocorticolimbic DAergic system of RLA and RHA rats [42] may be involved, at least in part, in their different responses of the BDNF/trkB system to FS observed in the present study.

## 4. Materials and Methods

### 4.1. Animals

Male outbred Roman rats (N = 32 for each line) were used throughout and were four-months-old (weight = 400–450 g) at the beginning of the experiments.

Animals were housed in groups of four per cage and maintained under temperature- and humidity-controlled environmental conditions (23 °C ± 1 °C and 60% ± 10%, respectively), with a 12 h light–dark cycle (lights on at 8:00 a.m.). Standard laboratory food and water were available *ad libitum*. To avoid stressful stimuli resulting from manipulation, the maintenance activities in the animal house were carried out by a single attendant and bedding in the home cages was not changed on the two days preceding the test. All procedures were performed according to the guidelines and protocols of the European Union (Directive 2010/63/EU). The experimental protocol was approved by the Committee for Animal Experimentation of the Universidad Autónoma de Barcelona and was authorized by the Ministerio de Ciencia e Innovación (authorization No. PID2020-114697GB-I00, 1 January 2021). Every possible effort was made to minimize animal pain and discomfort and to reduce the number of experimental subjects.

### 4.2. FS and Behavioral Measurements

The RHA and RLA rats were randomly assigned to the control or FS groups and were processed in parallel, according to a schedule that was counterbalanced for animal line and treatment. All animals (N = 64) were naive at the beginning of the experiments and were used only once. Rats in the FS groups (N = 16 for each line) were singly moved from the animal house to a sound-attenuated, dimly illuminated test room whereas the controls (N = 16 for each line) were kept in their home cages in the animal house until sacrifice. All testing was performed between 10:00 a.m. and 6:00 p.m. and consisted of a 15 min session of acute forced swimming, according to the experimental conditions previously described [19]. Briefly, rats were placed individually in plastic cylinders (58 cm tall × 32 cm diameter) which were filled with water at 24–25 °C to a 40-cm depth to ensure that they were unable to touch the bottom of the cylinder with their tails or hind paws. At the end of the 15 min swimming sessions, rats were removed from the cylinders, gently dried with paper towels, placed in a heated cage for 15 min, and singly transferred to an adjacent room where they were sacrificed. The water in the cylinders was replaced before starting the next test session. All the behaviors were quantified by a single well-trained observer who was blind to rat line. A time-sampling technique was used to record the predominant behavior in each 5 s period of the FS session [19]. The following behaviors were recorded: (1) Immobility—floating passively in the water without struggling and doing only those movements necessary to keep the head above water; (2) Immobility latency—the time from the beginning of the test until the first immobility episode; (3) Swimming—showing moderate active motions all around in the cylinder, more than necessary to simply keep the head above water; (4) Climbing—making active vigorous movements with the forepaws in and out of the water, usually directed against the walls; (5) Diving—swimming under water looking for a way out of the cylinder; and (6) Boli—number of fecal boli excreted. The behaviors were recorded only in a representative sample of animals from the control and FS groups that were subsequently used for the western blot (four rats from each line) or immunohistochemical assays (four rats from each line).

### 4.3. Sampling

Forty-five minutes after the end of the FS session, the animals used for the WBs (N = 32) were killed by decapitation whereas the animals used for the immunohistochemical assays (N = 32) were deeply anesthetized with chloral hydrate (500 mg/kg, i.p., 2 mL/kg) and a few minutes later were transcardially-perfused with ice-cold PBS (Phosphate Buffered Saline: 137 mM NaCl, 2.7 mM KCl, 10 mM Na2HPO4, 2 mM KH2PO4, pH 7.3) and 4% paraformaldehyde (PFA).

**Figure 13:**
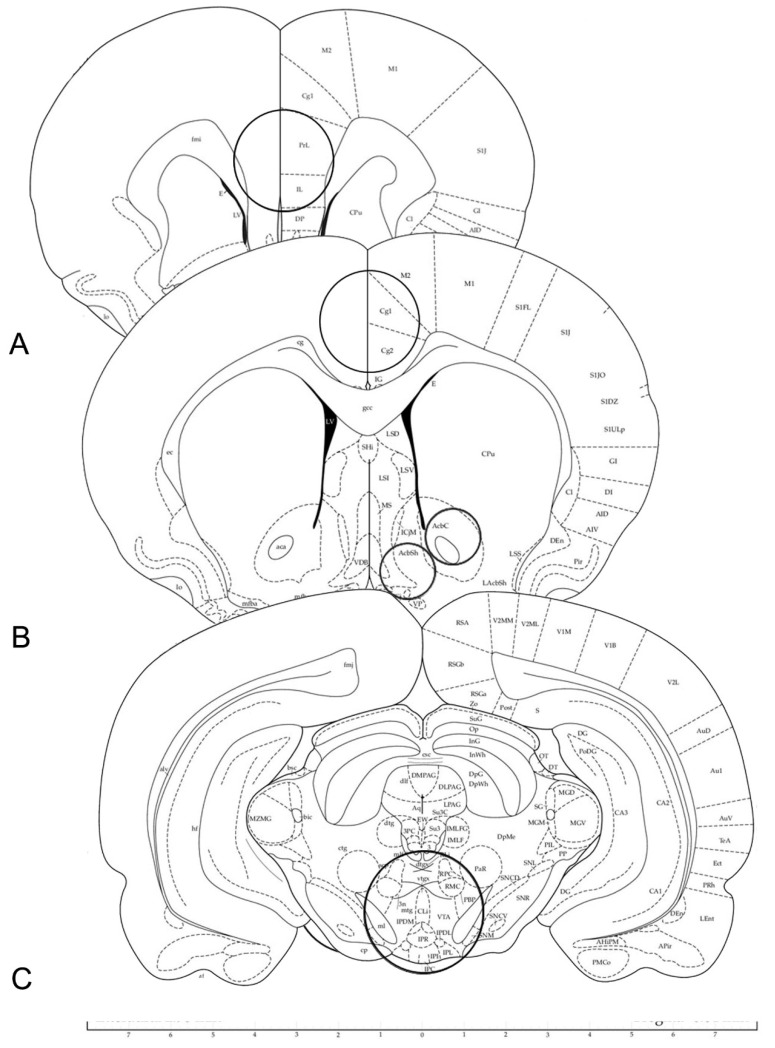
Schematic representation of rat brain coronal sections (plates Nr. 10, 14 and 44 adapted from Ref. [54]. Copyright © 1997 by George Paxinos and Charles Watson). The circles in (**A**–**C**) denote the areas of infra-limbic (IL) and pre-limbic (PL) regions of the medial prefrontal cortex (**A**); the cingulate cortex, the shell (AcbSh) and core (AcbC) subregions of the nucleus accumbens (**B**); the ventral tegmental area (VTA) (**C**); taken for western blot analysis using micropunches of 2.5 mm (**A**), 2.5 mm and 1.5 mm (**B**), and 3 mm (**C**).

The brains were rapidly removed from the skull immediately after sacrifice for the WB assays or after perfusion for the immunohistochemistry experiments. For WB, the brains were cooled in dry ice for 15 s, placed in a brain matrix, and cut in 2 mm thick coronal slices, using the stereotaxic coordinates of the rat brain atlas of Paxinos and Watson [54] as a reference. The anterior-posterior coordinates (from bregma) were approximately 3.70 mm for the PL/IL cortex, 1.7 mm for the ACg cortex and the Acb, and −5.8 mm for the VTA territory, respectively (Figure 13). The specimens for WB were taken by means of either bilateral or single (across the midline) micropunches with the following diameters: 2.5 mm for the PL/IL and ACg cortices; 1.5 mm for the Acb core and the Acb shell; 3 mm for the VTA [55] (Figure 13), The tissue punches were rapidly frozen at −80 °C and homogenized in distilled water containing 2% sodium dodecyl sulfate (SDS) (300 μL/100 mg of tissue) and a cocktail of protease inhibitors (cOmpleteTM, Mini Protease Inhibitor Cocktail Tablets, Cat# 11697498001, Roche, Basel, Switzerland). For immunohistochemistry, the brains were post-fixed by immersion in a freshly prepared 4% phosphate-buffered PFA, pH 7.3, for 4–6 h at 4 °C, and then rinsed until they sank in 0.1 M phosphate buffer (PB), pH 7.3, containing 20% sucrose.

### 4.4. Western Blot

Total protein concentrations were determined as described by Lowry et al. [56], using bovine serum albumin as a standard. Proteins from each tissue homogenate (40 μg), diluted 3:1 in 4× loading buffer (NuPAGE LDS Sample Buffer 4×, Cat# NP0008, Novex by Life Technologies, Carlsbad, CA, USA), were heated to 95 °C for 7 min, and separated by sodium dodecyl sulfate (SDS)-polyacrylamide gel electrophoresis (SDS-PAGE), using precast polyacrylamide gradient gel (NuPAGE 4–12% Bis-Tris Gel Midi, Cat# NP0321, Novex by Life Technologies, Carlsbad, CA, USA), in the XCell4 Sure LockTM Midi-Cell chamber (Life Technologies). Internal mw. standards (Precision Plus Protein Western C Standards, Cat# 161-0376, Bio-Rad, Hercules, CA, USA) were run in parallel. Blots were blocked by immersion in 20 mM Tris base and 137 mM sodium chloride (TBS), containing 0.1% Tween 20 (TBS-T) and 5% milk powder, for 60 min, at room temperature. The primary antibodies were rabbit polyclonal antibodies against BDNF (Cat# N-20 sc-546, RRID: AB_630940, Santa Cruz Biotechnology, Dallas, TX, USA) and trkB (Cat# (794) sc-12, RRID: AB_632557, Santa Cruz Biotechnology), both diluted 1:1000 in TBS containing 5% milk powder and 0.02% sodium azide. Incubations with primary antiserum were carried out for two nights at 4 °C. After rinsing in TBS/T, blots were incubated at room temperature, for 60 min, with a peroxidase-conjugated goat anti-rabbit serum (Cat#9169, RRID:AB_258434, Sigma Aldrich, St Louis, MO, USA), diluted 1:10,000 in TBS/T. Controls for equal-loading of the wells were obtained by immunostaining the membranes, as above, using a mouse monoclonal antibody against glyceraldehyde-3-phosphate dehydrogenase (GAPDH) (MAB374, RRID:AB_2107445, EMD Millipore, Darmstadt, Germany), diluted 1:1,000, as the primary antiserum, and a peroxidase-conjugated goat anti-mouse serum (AP124P, RRID:AB_90456, Millipore, Darmstadt, Germany), diluted 1:5,000, as the secondary antiserum. To control for non-specific staining, blots were stripped and incubated with the relevant secondary antiserum. In order to check for antibody specificity and cross-reactivity, the anti-BDNF antibody was challenged with 200 ng of rhBDNF (Cat# B-257, Alomone Labs, Jerusalem, Israel) [15]. After rinsing in TBS/T, protein bands were developed using the Western Lightning Plus ECL (Cat# 103001EA, PerkinElmer, Waltham, MA, USA), according to the protocol provided by the manufacturer, and visualized using the ImageQuant LAS-4000 (GE Healthcare, Little Chalfont, UK). Approximate molecular weight (mw) and relative optical density (O.D.) of the labeled protein bands were evaluated by a blinded examiner. The ratio of the intensity of the BDNF-positive and trkB-positive bands to the intensity of the GAPDH-positive ones was used to compare the relative expression levels of these proteins in both rat lines. The O.D. was quantified by the Image Studio Lite Software (RRID:SCR_014211, Li-Cor).

### 4.5. Immunohistochemistry

Coronal brain sections from the RLA and RHA rats were examined in pairs placed on the same slide. Semiconsecutive cryostat sections (14 μm thick) were collected on chrome alum-gelatin coated slides and processed by the avidin–biotin–peroxidase complex (ABC) immunohistochemical technique. The endogenous peroxidase activity was blocked with 0.1% phenylhydrazine (Cat# 101326606, Sigma Aldrich, St Louis, MO, USA) in phosphate-buffered saline (PBS), containing 0.2% Triton X-100 (PBS/T), followed by incubation with 20% of normal goat serum (Cat# S-1000, Vector, Burlingame, CA, USA). The primary antibodies (i.e., rabbit polyclonal antibodies against BDNF and trkB) were the same as those used for WB and both were diluted 1:500. A biotin-conjugated goat anti-rabbit serum (BA-1000, RRID: AB_2313606, Vector, Burlingame, CA, USA), diluted 1:400, was used as secondary antiserum. The reaction product was revealed with the ABC (Cat#G011-61, BioSpa Div. Milan, Italy), diluted 1:250, followed by incubation with a solution of 0.1 M PB, pH 7.3, containing 0.05% 3,3′-diaminobenzidine (Sigma Aldrich, St Louis, MO, USA), 0.04% nickel ammonium sulfate and 0.01% hydrogen peroxide. All antisera and the ABC were diluted in PBS/T. Incubation with primary antibodies was carried out overnight at 4 °C. Incubations with secondary antiserum and ABC lasted 60 min and were performed at room temperature. Negative control preparations were obtained by incubating tissue sections in parallel with either PBS/T, alone, or in one of the following ways: (i) with the relevant primary antiserum pre-absorbed with an excess of the corresponding peptide antigen (Cat# sc-546P and sc-12 P, for BDNF and trkB, respectively, Santa Cruz Biotechnology, Santa Cruz, CA, USA), or (ii) by substituting the corresponding primary antiserum with normal goat serum. Slides were observed with an Olympus BX61 microscope and digital images were captured with a Leica DFC450C camera.

### 4.6. Statistical Analyses

WB data were statistically evaluated by two-way ANOVA (see Table 2). Before performing the ANOVAs, data sets of each experimental condition were inspected for normal distribution of data and homogeneity of variances, with the Shapiro-Wilk’s test and the Bartlett’s test, respectively. Among the behavioral measurements, the diving dataset showed statistically significant unequal variances and, therefore, were analyzed with the Welch’s *t* test. Data sets that did not show homogeneity of variances, were log-transformed, and then analyzed by two-way ANOVA, as previously described [16,57]. When two-way ANOVAs revealed statistically significant interactions, the sources of significance were ascertained by pairwise *post-hoc* contrasts with the HSD Tukey’s test. In all the other cases, pair-wise comparisons were performed with the two-tailed *t* test with Sidak’s corrected alpha values. All the statistical analyses were carried out with the PRISM, GraphPad 6 Software (San Diego, CA, USA) with the significance level set at *p* < 0.05.

## 5. Conclusions 

This study shows that FS induced rapid and distinct changes in the levels of BDNF and trkB receptor proteins in different areas of the mesocorticolimbic system of RHA and RLA rats and displays the immunohistochemical localization of both proteins. Our results provide compelling evidence that the Roman rat lines represent a valid approach for studying the influence of the genetic background on BDNF/trkB signaling and the vulnerability or resilience to the prodepressant effects of stress. 

Preclinical and clinical studies indicate that a disruption in BDNF signaling alone is insufficient to elicit the constellation of depressive symptoms. On the other hand, it is well established that BDNF plays a role in the effects of both monoaminergic and fast-acting antidepressants, like ketamine, on synaptic plasticity, neuronal structure, and depression-like behavior [29,58,59]. However, the treatment with conventional monoaminergic antidepressants exerts its therapeutic action only after weeks or months, indicating that the increment in the CNS concentrations of serotonin, norepinephrine, or DA, which occur within minutes, is not directly responsible *per se* for the clinical actions of these drugs. Thus, because the BDNF-dependent plastic changes elicited by chronic treatment with monoaminergic antidepressants occur after several weeks, it has been proposed that the time course of the plastic effects of BDNF can explain the long latency of the clinical effects of monoaminergic antidepressants. On the other hand, the fast-acting antidepressant ketamine elicits a rapid release of BDNF and stimulation of trkB and Akt (protein kinase B), which then activates the intracellular signaling protein mTORC1 (mammalian target of rapamycin complex 1), leading to the rapid increase in the synthesis of proteins required for synapse maturation and formation [59].

Notably, BDNF in the HC produces effects that are opposite to those induced in the mesolimbic pathway in behavioral models of depression; therefore, caution should be exerted in developing therapies for depression having a single effect on BDNF all over the brain. Moreover, BDNF signaling is very complex, involving a series of parallel but interacting downstream cascades comprised of multiple subtypes of proteins, some of which show remarkable circuit-specific patterns of expression in the brain. Hence, it is not surprising that BDNF can exert both pro-depressive and antidepressant-like behavioral effects depending on the specific neural circuit in which BDNF/trkB signaling is regulated [29,31]. Furthermore, our results demonstrate also that the genetic background plays a key role in the regulation of BDNF/trkB signaling under basal conditions and after acute stress; thus, the same acute stressor impacts in opposite directions on BDNF/trkB transmission in the mesocorticolimbic system of depression-prone RLA rats and their depression-resilient RHA counterparts. Consequently, targeting drug development efforts towards agents that modulate synaptic plasticity, influence the BDNF/trkB signaling pathway, and restore synapse connectivity in a circuit-specific manner bears promise for the discovery of more effective antidepressants with innovative mechanisms of action.

Further studies in Roman rats aimed at identifying BDNF-related intracellular pathways and possibly different mutated variants of this trophin are warranted to enhance our understanding of the mechanisms promoting resilience or vulnerability under adverse situations. 

## Figures and Tables

**Table 1:** Behaviors displayed by RHA and RLA rats during the forced swimming session ^1^.

Behavioral Measures	RHA	RLA
Immobility latency (s)	291 ± 45	124 ± 21 *
Immobility time (s)	256 ± 19	538 ± 22 ***
Swimming (s)	299 ± 28	211 ± 36 *
Climbing (s)	286 ± 31	132 ± 12 ***
Diving (s)	59 ± 8	19 ± 2 ***
Fecal boli (n)	3 ± 2	7 ± 1 *

^1^ Shown are the mean ± SEM of eight rats in each experimental group. * *p* < 0.05; *** *p* < 0.001 vs. the RHA group (two-tailed Student’s *t* test for independent samples).

**Table 2:** F values and significance levels of two-way ANOVAs performed on WB data plotted in Figure 1, Figure 2, Figure 3, Figure 4, Figure 5 and Figure 6.

		Line		FS		Line × FS		
Brain Area	Marker	F Value	*p* Value	F Value	*p* Value	F Value	*p* Value	d.f.
VTA	BDNF	6.052	0.021	4.630	0.041	5.146	0.0318	1, 26
	trkB	2.764	ns	7.563	0.0103	5.794	0.0229	1, 28
Acb s	BDNF	0.055	ns	0.011	ns	2.684	ns	1, 28
	trkB	5.517	0.0261	0.1088	ns	0.0647	ns	1, 28
Acb c	BDNF	2.723	ns	2.984	ns	9.224	0.0051	1, 28
	trkB	1.717	ns	1.550	ns	14.49	0.0007	1, 28
ACg cortex	BDNF	3.266	0.0082	4.267	0.048	7.956	0.009	1, 28
	trkB	0.035	ns	0.009	ns	18,98	0.0002	1, 28
IL/PL cortex	BDNF	2.829	ns	0.548	ns	32.45	<0.0001	1, 28
	trkB	3.641	ns	6.481	0.0167	8.165	0.008	1, 28

ns = not significant.

## Data Availability

The data presented in the current study are available from the corresponding author on reasonable request.

## Abbreviations

ABC	avidin–biotin–peroxidase complex
Acb	nucleus accumbens
ACg	anterior cingulate
AMYG	amygdala
ANOVA	analysis of variance
BDNF	brain derived neurotrophic factor
CSDS	chronic social defeat stress
CUS	chronic unpredictable stress
DA	dopamine
dHC	dorsal hippocampus
FS	forced swimming
GAPDH	glyceraldehyde-3-phosphate dehydrogenase
HC	hippocampus
IL	infralimbic
LI	prelimbic
mw	molecular weight
mTORC1	mammalian target of rapamycin complex 1
O.D.	relative optical density
PBS	phosphate buffered saline
PFA	paraformaldehyde
PFC	prefrontal cortex
PL	prelimbic
RHA	roman high avoidance
RLA	roman low avoidance
SDS-PAGE	sodium dodecyl sulphate-polyacrylamide gel electrophoresis
TBS-T	tris base, sodium chloride, tween 2
trkB	tyrosine receptor kinase b
vHC	ventral hippocampus
VTA	ventral tegmental area
WB	western blot
